# Extracellular Vesicles: A New Perspective in Tumor Therapy

**DOI:** 10.1155/2018/2687954

**Published:** 2018-04-23

**Authors:** Yan-Zi Sun, Jun-Shan Ruan, Zong-Sheng Jiang, Ling Wang, Shao-Ming Wang

**Affiliations:** ^1^School of Pharmacy, Fujian Medical University, Fuzhou, China; ^2^Molecular Biology Laboratory of Traditional Chinese Medicine, Fujian Provincial Hospital, Clinical College of Fujian Medical University, Fuzhou, China

## Abstract

In recent years, the study of extracellular vesicles has been booming across various industries. Extracellular vesicles are considered one of the most important physiological endogenous carriers for the specific delivery of molecular information (nucleonic acid, cytokines, enzymes, etc.) between cells. It has been discovered that they perform a critical role in promoting tumor cell growth, proliferation, tumor cell invasion, and metastatic ability and regulating the tumor microenvironment to promote tumor cell communication and metastasis. In this review, we will discuss (1) the mechanism of extracellular vesicles generation, (2) their role in tumorigenesis and cancer progression (cell growth and proliferation, tumor microenvironment, epithelial-mesenchymal transition (EMT), invasion, and metastasis), (3) the role of extracellular vesicles in immune therapy, (4) extracellular vesicles targeting in tumor therapy, and (5) the role of extracellular vesicles as biomarkers. It is our hope that better knowledge and understanding of the extracellular vesicles will offer a wider range of effective therapeutic targets for experimental tumor research.

## 1. Introduction

Extracellular vesicles are small membrane vesicles (30–150 nm in diameter) of endocytic origin that are formed through the fusion of the plasma membrane with multivesicular endosomes (MVEs), and subsequently exocytosed [1]. Many cell types, including dendritic cells (DCs), T cells, macrophages, B cells, mastocytes, reticulocytes, active neurons, and tumor cells, can secrete extracellular vesicles inductively or constitutively [2]. They have also been found in human body fluids, such as blood and urine. Extracellular vesicles are secreted nanovesicles that play a key role in cell-cell communication by transferring nucleic acids and proteins to target cells and tissues. Extracellular vesicles content depends on their donor cell type. They can interact with stromal cells in the tumor microenvironment, which forms at sites of future metastases, to promote disseminated tumor cell survival and outgrowth and to increase tumor cell invasiveness. Moreover, they are involved in directionally transporting cargo to specific locations. Different cells derive extracellular vesicles with unique cargo, commonly miRNAs or integrins, which may be used as molecular markers in tumor diagnosis to determine the specific cancer subtype. Tumor biomarkers can also be utilized in cancer patient therapy and prognostics. The epithelial-mesenchymal transition (EMT) is the process through which epithelial cells change from an epithelial cobblestone phenotype to an elongated fibroblast phenotype. At present, the EMT is known to be involved in the movement of tumor cells to new areas. Extracellular vesicles are responsible for intercellular communication between tumor cells and other cells in the tumor microenvironment. Theoretically, extracellular vesicles should be involved in EMT and cell migration. In fact, there have been some studies linking extracellular vesicles to metastasis. This review will focus on the role of extracellular vesicles in cancer progression to explore their underlying mechanism, as well as their potential role as biomarkers in tumor diagnosis and prognosis. This will hopefully provide some theoretical basis and direction to extracellular vesicles research and clinical tumor treatment.

## 2. The Mechanism of Exosome Biogenesis

### 2.1. Extracellular Vesicles Secretion

Extracellular vesicles have been widely studied, but its mechanism of biogenesis is still unclear. There are many factors that can induce extracellular vesicles production. Fruhbeis found that glutamate triggers exosome release from differentiated oligodendrocytes by activating Ca2+-permeable glial NMDA and AMPA receptors [3]. Another study found that radiation treatment can stimulate increased extracellular vesicles release from T cells [4, 5]. Radiation can also stimulate glioma cells, prostate cancer cells, and lung cancer cells to release extracellular vesicles. However, radiation treatment did not change extracellular vesicles size or diameter [6]. Moreover, hypoxia is also an important factor in stimulating extracellular vesicles secretion. In short, all factors that can cause changes in the cellular environment can promote extracellular vesicles production. The Rab27 small GTPases, including Rab27a and Rab27b, have been reported to regulate extracellular vesicles biogenesis and secretion. In this study, they found that Rab27a and Rab27b have a key role in extracellular vesicles secretion; Rab27a and Rab27b silencing inhibits exosome secretion, by promoting the targeting of MVEs to the cell periphery and their docking at the plasma membrane [7]. Recently, site-directed mutagenesis and gene rescue studies showed that Akt-mediated activation of PRAS40 via threonine-246 phosphorylation is both necessary and sufficient to cause exosome secretion, without affecting the ER/Golgi pathway [8]. It is essential to find the key elements regulating extracellular vesicles secretion, as these are potential targets for cancer treatment and would be useful in the development of novel cancer therapies.

### 2.2. Extracellular Vesicles Sorting and Positioning

**Figure 1:**
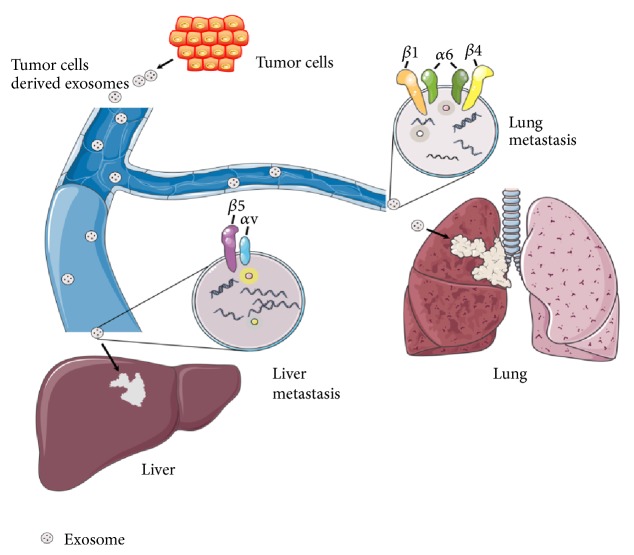
Integrins are a large family of heterodimeric transmembrane receptors that mediate cell attachment to other cells or to the extracellular matrix via interactions with proteins such as fibronectin and collagen. Integrins are heterodimers composed of an *α* and a *β* subunit, and they play important roles in many physiological and pathological processes. Yuwen et al. found that exosomal integrin *α*6*β*4 and integrin *α*6*β*1 were closely linked to lung metastasis, and integrin *α*V*β*5 was closely linked to liver metastasis.

Extracellular vesicles can be precisely targeted to specific cells, and its internalization is likely to depend on the properties of outer membrane proteins and lipids, especially those associated with the extracellular matrix and adhesion. Sterzenbach et al. present an elegant strategy for specific sorting of protein cargo to extracellular vesicles by engineering an extracellular vesicles address signal to the protein of interest [9]. The authors further demonstrate that, upon administration via the nasal route, extracellular vesicles carry the engineered protein across the blood-brain barrier and target its biological activity to neural cells. Cargo sorting during extracellular vesicles formation is known to involve endosomal sorting complex required for transport (ESCRT) proteins acting at the multivesicular body- (MVB-) limiting membrane [10]. While, to date, most strategies for linking biomolecules to extracellular vesicles have dealt with RNA or small molecules, a specific approach for active and selective protein packaging has, thus far, been lacking. Future studies could generate novel cancer treatments by interfering with or inhibiting novel targets, to reduce the release of specific extracellular vesicles, or by means of extracellular vesicles site-directed drugs, to reduce tumor cell growth, proliferation, and metastasis. Hoshino et al. found that extracellular vesicles act as a boat-like carrier to transport cargo between cells and can selectively load proteins and genetic material to reach specific target organs and cells [11]. They found that extracellular vesicles integrin *α*6*β*4 and integrin *α*6*β*1 were closely linked to lung metastasis, and integrin *α*V*β*5 was closely linked to liver metastasis. If we downregulate integrin *α*6*β*4 and integrin *α*V*β*5 expression, it could prevent lung and liver cancer metastasis (Figure 1). This indicates that extracellular vesicles membrane integrins play a key role in selective organ metastasis. This study provides another measure for the prediction and treatment of metastatic cancers and further supports the “seed and soil” theory of cancer metastasis, with extracellular vesicles functioning as the “seed.”

### 2.3. Extracellular Vesicles Uptake and Internalization

Extracellular vesicles fuse to recipient cells, releasing their cargo into the recipient cell, in a process of endocytosis. This process is dependent on receptor molecules on the recipient cell surface. It is important to find the splicing molecules involved in extracellular vesicles absorption and internalization so that this process can be targeted and interrupted. Furthermore, this would hinder extracellular vesicles communication, inhibiting tumor cell growth, proliferation, invasion, and metastasis. Heparan sulfate plays a dual role in extracellular vesicles-cell interaction [12]. On extracellular vesicles, heparan sulfate captures fibronectin and acts as a fibronectin receptor on target cells. Removal of heparan sulfate from the extracellular vesicles surface releases fibronectin and dramatically inhibits extracellular vesicles-target cell interaction. In pancreatic ductal adenocarcinoma (PDAC), the healthy pancreatic tissue surrounding the tumor releases REG3*β*, a lectin that binds the glycoproteins present on the surface of endocytic vesicles (EVs), thus interfering with extracellular vesicles uptake and internalization by target cells [13]. This provides a novel strategy for treating PDAC, and the feasibility of targeting REG3*β* should be studied further.

## 3. Extracellular Vesicles Regulate the Tumor Microenvironment

### 3.1. Extracellular Vesicles Regulate the Tumor Microenvironment for Tumor Cell Growth and Angiogenesis

The tumor microenvironment occupies a pivotal position in cancer. Extracellular vesicles carry their cargo to neighboring cells or distant organs. Tumor-derived extracellular vesicles are absorbed by other organs and tissues to create a suitable environment for tumor cell growth and proliferation, enhancing the invasiveness and metastatic ability of recipient cells. Some extracellular vesicles can also regulate cell apoptosis; for example, pancreatic cancer cell-derived vesicles induce cell apoptosis by regulating Bcl-2 protein expression and activating PTEN and Gsk-3*β*, blocking the PI3K/Akt signaling pathway [14]. Extracellular vesicles mostly act as cancer cell accomplices to harm human health. Immune cells within the tumor microenvironment, such as tumor-associated macrophages, promote tumor growth and their action has also been connected to tumor invasion, metastasis, and angiogenesis [12]. This behavior has been attributed to intercellular communication, driven in part by tumor cell-secreted vesicles. VEGF and EGFR are proteins critical to angiogenesis and have increased expression in mutant extracellular vesicles [15]. Elevated levels of VEGF and EGFR may be transferred to recipient cells to enhance angiogenesis, which is necessary for tumor growth. Angiogenesis is also a key process in the preparation of lymph nodes for melanoma metastasis. Melanoma extracellular vesicles induce GM-CSF expression by endothelial cells in vitro and HIF-1*α* expression in premetastatic lymph nodes in vivo [16]. Melanoma extracellular vesicles can stimulate SCS M1 or M2 M*φ* function by endothelial-derived GM-CSF in lymph nodes, which may induce different but complementary protumor angiogenic processes.

### 3.2. Extracellular Vesicles Regulate the Tumor Microenvironment for EMT and Migration

Metastasis is a basic biological feature of malignant tumors, and 90% of cancer deaths result from metastatic tumors. EMT is the process of epithelial cell transformation to mesenchymal cells. During this process, cells lose cell polarity and decrease expression of adhesion proteins, such as epithelial cadherin (E-cadherin) and tight junction protein (ZO-1), intercellular connection loosens, and there is intracellular skeletal protein recombination. These changes lead to decreased adhesion between cells and increased migration capacity, making it easier for tumor cells to leave their original location and infiltrate and settle in new tissue to form distal tumors. Extracellular vesicles content reflects changes in the cell phenotype. Thrombin-activated, platelet-releasing exosomes transfer miRNA-223 between cells [17]. Further studies have shown that miRNA-223 downregulates adhesion molecule expression, including ICAM-1, to promote EMT by interfering with NF-kB and the MAPK pathway. Several studies have linked extracellular vesicles to metastasis. In the transplanted breast cancer model, nSMase2 knockout can reduce extracellular vesicles secretion and significantly reduce tumor metastasis [18]. Jeppesen et al. have identified several protein changes in the membrane and lumen of bladder cancer cell-derived extracellular vesicles that are related to increased metastatic propensity [19]. Several of the proteins with an increased abundance are related to EMT. Kim et al. investigated the changes in extracellular vesicles cargo from the epithelial to mesenchymal cell phenotype by inducing EMT with transforming growth factor- (TGF-) *β*1 in A549 human lung adenocarcinoma cells [20]. In vitro coculture of tumor-associated macrophage- (TAM-) derived extracellular vesicles with endothelial cells suppressed endothelial cell migration [21]. However, when epithelial ovarian carcinoma- (EOC-) derived extracellular vesicles were added into the coculture system, the migration of endothelial cells was restored, indicating that EOC-derived exosomes play a central role in regulating the interaction between TAMs and endothelial cells. There are many studies demonstrating that extracellular vesicles play an important role in tumor invasion and migration. We will need to determine extracellular vesicles mechanisms in different tumor tissue types, to provide a strategic approach to fighting cancer.

## 4. The Role of Extracellular Vesicles in Immune Therapy

Studies have shown that extracellular vesicles can promote the immune system, highlighting a new potential strategy for cancer vaccine research. Extracellular vesicles promote the immune system by carrying cargo, such as miRNAs, cytokines, and proteins, to regulate the immune response. In cancer research, most of the previous studies were to explore cancer cells derived exosomes but rarely understood the function of normal cells derived extracellular vesicles. Natural killer (NK) cells have a rapid immune response to metastatic or hematological malignancies and have been clinically developed for antitumor properties of NK cells. However, the characteristics and function of NK cells derived extracellular vesicles are still unknown. The Byeong-Cheol Ahn study group from Gyeongbuk National University School of Medicine, Korea, explored the antitumor effects of NK cell derived extracellular vesicles mediated antimelanoma in vivo and in vitro [22]. This study found that NK cell derived extracellular vesicles are cytotoxic to melanoma cells but have no effect on normal cells. FasL inhibitors can reduce its cytotoxicity to melanoma cells. NK cell derived extracellular vesicles induce apoptosis of melanoma cells in vitro. The results of this study show that NK cell derived extracellular vesicles produce cytotoxic effects on melanoma cells and deserve further development of potential immunotherapy strategies for cancer. Huang et al. found that exosomes from TGF-*β*1-silenced L1210 cells (LEXTGF-*β*1si) can enhance the efficacy of dendritic cell- (DC-) based vaccines by decreasing TGF-*β*1 in DCs, effectively promoting their maturation and immune function [23]. MiRNAs can regulate gene expression, controlling many cellular functions such as proliferation, differentiation, apoptosis, oncogenesis, and drug sensitivity in tumor cells [24]. The role of miRNAs in regulating the balance between macrophage M1 and M2 phenotypes, as well as impacting the recruitment of other immune cells in the tumor microenvironment, is well established. Along with modulated wt-p53 and miR-125b expression, these extracellular vesicles possess a reprogramed global miRNA profile and active apoptotic and p53-signaling pathways [25]. Furthermore, an altered miRNA profile also mediates macrophage repolarization towards a more proinflammatory/antitumor M1 phenotype. Antigen presenting cell- (APC-) derived extracellular vesicles carry tumor antigens in vivo, which can inhibit tumor cell growth by stimulating CD4+ and CD8+ T cells [26]. Extracellular vesicles can also inhibit the immune response, which may be useful in the investigation and treatment of autoimmune diseases. For example, extracellular vesicles in conditional plasma sera derived from immunosuppressive dendritic cells show a significant effect in animal inflammation and autoimmune diseases [27]. Except for immune cells, mesenchymal stem cells (MSCs) have been widely used for tissue repair and regeneration, because of their immunosuppressive and anti-inflammatory effects. Another study found that extracellular vesicles isolated from MSCs can promote the proliferation and differentiation of human primary osteoblastic cells (HOBs) and have potential use in bone tissue regeneration [28]. Not only does extracellular vesicles cargo regulate the immune response, but also extracellular vesicles membrane-associated protein can be used as a therapeutic. CD47, a “do not eat me” signal, is overexpressed on the surface of most tumors and interacts with signal-regulatory protein *α* (SIRP*α*) on phagocytic cells. By engaging SIRP*α*, CD47 limits the ability of macrophages to engulf tumor cells, acting as a major phagocytic barrier. The SIRP*α*-extracellular vesicles-mediated CD47 blockade causes tumor growth inhibition. Koh et al. developed an extracellular vesicle-based immune check point blockade that antagonizes the interaction between CD47 and SIRP*α*. Tumors receiving SIRP*α*-extracellular vesicles treatment showed extensive CD8+ T cell infiltration [29]. Intensive infiltration of CD8+ T cells indicates that SIRP*α*-extracellular vesicles could successfully trigger a local immune response at the tumor microenvironment, resulting in tumor regression.

## 5. Extracellular Vesicles Trafficking in Tumor Therapy

Extracellular vesicles are the size of a virion and are released by all cells. It is therefore naturally present in the blood and can thus easily migrate to and enter target cells in vivo. Extracellular vesicles are thus viewed as ideal natural carriers for the transport of drugs and genes. Pascucci et al. treated interstitial cells with paclitaxel and subsequently detected paclitaxel in cell supernatant extracellular vesicles. Moreover, extracellular vesicles containing paclitaxel could inhibit tumor cell growth in vitro [30]. It has been shown that treating cancer cells with different doses of curcumin lead to the release of extracellular vesicles containing curcumin [31]. These extracellular vesicles could induce anticancer effects in recipient cells and reduce tumor growth. Since extracellular vesicles can carry a variety of different molecules, modified extracellular vesicles could be applied as a novel cancer drug-delivery method. This would require appropriate donor cells, capable of secreting a large number of extracellular vesicles, that do not generate an inflammatory response (e.g., mature dendritic cells or other APCs can secrete extracellular vesicles with immunoreactive substances) and are stable for a sufficiently long enough time to transport their goods to target organs [32]. A recent study utilized genetically modified extracellular vesicles to directly and specifically target KRAS mutations frequently associated with pancreatic cancer [33]. In this study, genetically modified extracellular vesicles (referred to as iExosomes) were able to transport small RNA molecules specifically targeting the mutant KRAS gene, leading to alleviation of murine pancreatic cancer and increasing overall survival rate. These researchers used a targeted method called RNA interference (RNAi), using natural nanoparticles (exosomes) to transport small interfering RNA (siRNA) or short hairpin RNA (shRNA) molecules to target KRAS mutations. Studies have confirmed that exosomes can function as an efficient RNAi vector. We could use the same method to treat different tumors. If the tumorigenic mutant gene is known, then modified exosomes carrying targeted RNAi molecules for the mutant gene to the specific tumor site should have a curative effect.

## 6. Extracellular Vesicles as Biomarkers

Tumor-derived extracellular vesicles have emerged as promising cancer biomarkers due to their availability in biological fluids, high stability, and capability of representing parent cells and associated roles in critical physiological activities, including transport of oncoproteins and tumor-specific RNA molecules throughout the body. As such, they are considered highly sensitive, minimally invasive biomarkers for cancer diagnosis and prognosis. Yuwen et al. have indicated that advanced non-small-cell lung carcinoma (NSCLC) patients with low serum extracellular vesicles miR-146a-5p levels had higher recurrence rates than those with high levels [34]. Serum extracellular vesicles miR-146a-5p may be a new biomarker predicting the efficacy of cisplatin for NSCLC patients. Dejima et al. found that plasma extracellular vesicles miR-21 and mir-4257 expression have potential as a predictive biomarker for recurrence in NSCLC patients who have received curative resection [35]. Another study demonstrated circulating plasma extracellular vesicles miR-125a-3p are readily accessible as a diagnostic biomarker for early-stage colon cancer [36]. Increased risk of liver metastasis and later colorectal cancer (CRC) TNM stage are associated with decreased miR-638 levels in serum extracellular vesicles, which may serve as disease biomarkers and novel therapeutic targets for CRC [37]. These studies have shown that extracellular vesicles can be used to monitor disease progression. Not only identifying that tumor-derived extracellular vesicles biomarkers are able to distinguish between adenocarcinoma (AC) and squamous cell carcinoma (SCC) can be used as a noninvasive method in the early diagnosis of NSCLC. Jin et al. indicated that miR-181-5p, miR-30a-3p, miR-30e-3p, and miR-361-5p were AC-specific, and miR-10b-5p, miR-15b-5p, and miR-320b were SCC-specific [38]. Tumor-derived extracellular vesicles miRNAs were observed by next-generation sequencing, and their diagnostic accuracy was verified. These miRNAs may be promising and effective candidates in the development of highly sensitive, noninvasive biomarkers for early NSCLC diagnosis. Hoshino et al. found tumor extracellular vesicles integrins determine organotropic metastasis [11]. They determined that extracellular vesicles integrin *α*6*β*4 and integrin *α*6*β*1 were closely linked to lung metastasis, and integrin *α*V*β*5 was closely linked to liver metastasis. This indicates that integrins can also function as predictive biomarkers of tumor development. We expect further discoveries regarding the role of extracellular vesicles as tumor biomarkers in the future, providing greater means for monitoring tumor progression, diagnosis, and prognosis (Table 1).

## 7. Conclusion 

There is growing evidence that extracellular vesicles play an irreplaceable and vital role in tumorigenesis. Extracellular vesicles studies have also provided us with novel strategies for diagnosing and treating tumors. In tumor diagnosis, extracellular vesicles can be used as special biomarkers. In tumor treatment, extracellular vesicles can be targeted in the following ways: obstruct their release from donor cells, preventing them from creating a tumor environment; knock out or close the key extracellular vesicles membrane protein to hinder extracellular vesicles positioning and secretion; or create mutant gene modifying extracellular vesicles, which exploit extracellular vesicles traffic to treat disease. Studies have also indicated that extracellular vesicles cargo can reverse tumor drug resistance. For example, miR-146a-5p overexpression was found to reverse resistance in cisplatin-resistant lung adenocarcinoma cells (A549/DDP) [34]. The role of extracellular vesicles in the treatment of cancer is self-evident. What is needed now is greater research into the function of extracellular vesicles in tumor cells, which will not only help us to understand tumor development, but also prove being beneficial to tumor diagnosis, treatment, and prognosis.

## Figures and Tables

**Table 1:** Extracellular vesicles cargo as the biomarker of diseases.

Diseases	Extracellular vesicles biomarkers
Advanced melanoma	miR-125b
Melanoma	miR-17/miR-19a/miR-21/miR-126/miR-149
Esophageal cancer recurrence distant metastasis	miR-21
Ovarian	EpCAM
Lung metastasis	Integrin *α*6*β*4 Integrin *α*6*β*1
Liver metastasis	Integrin *α*5*β*1
MET oncoprotein IV melanoma	TYRP-2/VLA-4/Hsp-70
Melanoma	Cavedin-1/S100B
Prostate cancer MET	miR-141/miR-375
Liver metastasis	Melan-A
Pancreatic cancer	Glypican-1
Pancreatic ductal adenocarcinoma	MIF
Colon cancer recurrence	miR-17-92a
Poor per-prognosis	miR-19a
Castration resistant tumor	miR-1290/miR-375
The presence of metastasis	miR-141
